# Autofluorescence-Based Identification and Functional Validation of Antennal Gustatory Sensilla in a Specialist Leaf Beetle

**DOI:** 10.3389/fphys.2019.00343

**Published:** 2019-03-28

**Authors:** Stefan Pentzold, Frédéric Marion-Poll, Veit Grabe, Antje Burse

**Affiliations:** ^1^Max Planck Institute for Chemical Ecology, Jena, Germany; ^2^UMR Evolution, Génomes, Comportement, Ecologie, CNRS, IRD, Univ Paris-Sud, Université Paris-Saclay, Gif-sur-Yvette, France; ^3^AgroParisTech, Université Paris-Saclay, Paris, France

**Keywords:** contact chemosensation, *sensilla chaetica*, cuticular autofluorescence, gustation, herbivory, antenna, leaf beetle, *Chrysomela populi*

## Abstract

Herbivorous insects mainly rely on their sense of taste to decode the chemical composition of potential hosts in close range. Beetles for example contact and scan leaves with their tarsi, mouthparts and antennal tips, i.e., appendages equipped with gustatory sensilla, among other sensillum types. Gustatory neurons residing in such uniporous sensilla detect mainly non-volatile compounds that contribute to the behavioral distinction between edible and toxic plants. However, the identification of gustatory sensilla is challenging, because an appendage often possesses many sensilla of distinct morphological and physiological types. Using the specialized poplar leaf beetle (*Chrysomela populi*, Chrysomelidae), here we show that cuticular autofluorescence scanning combined with electron microscopy facilitates the identification of antennal gustatory sensilla and their differentiation into two subtypes. The gustatory function of *sensilla chaetica* was confirmed by single sensillum tip-recordings using sucrose, salicin and salt. Sucrose and salicin were found at higher concentrations in methanolic leaf extracts of poplar (*Populus nigra*) as host plant compared to willow (*Salix viminalis*) as control, and were found to stimulate feeding in feeding choice assays. These compounds may thus contribute to the observed preference for poplar over willow leaves. Moreover, these gustatory cues benefited the beetle’s performance since weight gain was significantly higher when *C. populi* were reared on leaves of poplar compared to willow. Overall, our approach facilitates the identification of insect gustatory sensilla by taking advantage of their distinct fluorescent properties. This study also shows that a specialist beetle selects the plant species that provides optimal development, which is partly by sensing some of its characteristic non-volatile metabolites via antennal gustatory sensilla.

## Introduction

Plant-feeding insects often judge the suitability of potential hosts as food source or oviposition site by sensing various phytochemicals (Van Naters and Carlson, 2006; Agnihotri et al., 2016). While the olfactory system serves in attraction at a distance by identifying mainly volatiles, the gustatory system acts in close range by decoding mainly non-volatile phytochemicals (Chapman, 2003). Thus, gustation (taste, contact chemosensation) is used by phytophagous insects to determine host quality and initiate feeding (Ômura, 2018).

Upon arrival at the leaf surface, chrysomelid leaf beetles contact and scan leaves by extensive antennation, palpating and test biting, which constitutes a critical stage to final plant acceptance and rejection (Mitchell et al., 1999). The antennae, mouthparts and tarsi are equipped with contact gustatory sensilla, i.e., tiny cuticular, hair-like structures that house gustatory receptor neurons (GRNs) (Hallem et al., 2006; Zhang et al., 2016; Faucheux and Kundrata, 2017). Morphologically, insect gustatory sensilla have an apical pore (Isidoro et al., 1998; Hallberg and Hansson, 1999) allowing non-volatile phytochemicals to enter and dissolve into the aqueous sensillum lymph to reach and stimulate GRNs, ultimately leading to behavioral responses. However, since one appendage usually possesses a high number and different types of sensilla each comprising different sensory functions (e.g., chemo-, mechano-, hygro- thermosensory) (Hallberg and Hansson, 1999), the identification of taste sensilla is often challenging. Usually, scanning or transmission electron microscopy is used to identify putative pores and their position on each sensillum (Faucheux and Kundrata, 2017; Zhang et al., 2017). Taking advantage of distinct fluorescent properties of different sensilla types may provide the potential to distinguish among them by using fluorescence microscopy, which has so far mainly been tested on tarsal setae of ladybird beetles (Peisker et al., 2013). Nevertheless, additional electrophysiological recording analyses are required for functional characterisation of gustatory sensilla (e.g., Sollai et al., 2017).

Suitable host plants are chemically characterized by possessing compounds that act as behavioral stimulants and lacking those that act as behavioral deterrents (Ômura, 2018). Whereas plant primary metabolites such sucrose are often detected by antennal GRNs and thus stimulate feeding of different insect species, plant secondary metabolites are usually deterrent, growth inhibiting or even toxic to generalist insect herbivores (Isidoro et al., 1998; Chapman, 2003; Osier and Lindroth, 2006; Jørgensen et al., 2007; Alabi et al., 2014). Interestingly, the same secondary metabolites often stimulate feeding of specialists that have overcome and may even exploit host chemical defense (Bernays et al., 2000; Robert et al., 2012; Gray et al., 2015; Müller et al., 2015; Sollai et al., 2017). This pattern has been found for different insect species in case of salicinoids, which are phenolic glucosides typically found in Salicaceae plants (Fernandez and Hilker, 2007; Boeckler et al., 2011; Julkunen-Tiitto and Virjamo, 2016). One such intriguing example is the specialized poplar leaf beetle *Chrysomela populi* that specifically feeds on leaves of poplar species (*Populus* ssp.), an economically important tree known to contain various salicinoids (Gruppe et al., 1999; Boeckler et al., 2011). *C. populi* sequesters salicin for use in its own defense (Pasteels et al., 1983; Burse et al., 2009); however, it is unclear if and how non-volatile host salicinoids are tasted by the beetle, and if host primary metabolites such as sucrose or glucose play an additional role in host identification.

Here we take advantage of the distinct fluorescent properties of the different sensilla (sub)types on the antennae of a specialized phytophagous beetle and combine confocal laser scanning and electron microscopy, which facilitates identification of gustatory sensilla. The gustatory function of these *sensilla chaetica* was confirmed by electrophysiological tip recordings using sugars such as sucrose and salicin. Finally, we show that adult *C. populi* select a certain plant species which serves optimal weight gain and is partly due to tasting some of the host’s characteristic metabolites.

## Materials and Methods

### Biological Material

Poplar leaf beetles (*C. populi*, Linnaeus, Chrysomelidae, Coleoptera, Insecta) were collected in the north of Jena close to Dornburg-Camburg (+51°00′52^′′^, +11°38′17^′′^), Germany and reared in a climate chamber provided with fresh twigs of poplar under a 16 h:8 h light:dark cycle at 20°C. For experiments, beetles between three and 15 days old were used. Poplar (*Populus nigra*) and willow (*Salix viminalis*) plants, both species belonging to the Salicaceae family, were grown in the institute’s green house. Leaves of *P. nigra* are usually considered as main host of *C. populi*, whereas *S. viminalis* leaves are preferred by other leaf beetle species such as *Chrysomela saliceti*, Weise and *Plagiodera versicolora* (Laicharting) (Gök and Çilbiroğlu, 2005; La Spina et al., 2010).

### Fluorescence Imaging

Antennae were dissected from cold anesthetized adults of *C. populi* (*n* = 4), briefly cleaned in an Ultrasonic Cleaner (VWR^TM^) and mounted in glycerol (G9012 Sigma-Aldrich) in two cover slips, allowing microscopic observation of the distal antennal tip from both sides. Autofluorescence scanning images were acquired on a confocal laser scanning microscope (LSM 880, Zeiss, Oberkochen, Germany) using a 40×/1.2 W C-Apochromat. Excitation of cuticular autofluorescence was conducted by a 405 nm laser diode and the fitting main beam splitter 405. Emission was detected in Lambda mode between 410 and 695 nm to discriminate between high and low concentrations of resilin or chitin, respectively. Approximately 300 scans at a thickness of 0.5 μm were employed to image the terminal (9th) flagellomere of the antennae from both sides. Maximum intensity projections were used for visualization of the entire image stacks. Images were processed using ZEN software (Zeiss) and ImageJ (Schneider et al., 2012).

### Electron Microscopy

Scanning electron microscopy was employed to identify morphological details of antennal sensilla, e.g., an apical pore. Three days old beetles were fixed with 2.5% (v/v) glutaraldehyde in cacodylate buffer for 60 min. Afterwards the samples were washed three times for 10 min with cacodylate buffer and dehydrated in ascending ethanol concentrations (30, 50, 70, 90, and 100%) for 20 min each. Subsequently, the samples were critical-point dried using liquid CO_2_ and sputter coated with platinum (thickness approximately 8 nm) using a CCU-010 sputter coater (safematic GmbH, Bad Ragaz, Switzerland) to avoid surface charging. Finally, the specimens were investigated with a field emission scanning electron microscope LEO-1530 Gemini (Carl Zeiss Microscopy GmbH, Jena, Germany) at 8–12 kV.

### Electrophysiological Recordings

Electrophysiological recordings were obtained from GRNs in *s. chaetica* subtypes 1 and 2 found on the terminal flagellomere of adult *C. populi* beetles using single sensillum tip recordings (Hodgson et al., 1955). Live beetles were immobilized on a microscope slide using adhesive pads (UHU^®^ Patafix, Bolton Adhesives). The recording electrode (a thin-walled borosilicate glass capillary, World Precision Instruments) was pulled in a two-step electrode puller (PP-830, Narishige Group, Japan) to a tip diameter of approximately 5 μm. The recording electrode containing the test solution was placed over single sensilla during 2–3 s using a motorized piezo-micromanipulator. The following tastants, all freshly dissolved in 30 mM KCl and obtained from Sigma-Aldrich, were used: sucrose (1, 100 and 300 mM; *n* = 9 insects), fructose (100 and 300 mM; *n* = 4 insects) and salicin (100 mM, *n* = 2); as positive control 30 mM KCl were used (*n* = 3). Each stimulus was applied twice, i.e., with a time interval of 30 s to avoid adaptation. The grounded reference electrode was a 1 mm diameter silver chloride wire placed into the beetle’s abdomen and moved carefully as close as possible to the head and antennae. The recording electrode was connected to a TastePROBE amplifier [10×, Syntech (Marion-Poll and Van Der Pers, 1996)] and the signals were further amplified and filtered (high pass 10 Hz, low pass 3000 Hz) using a CyberAmp 320 amplifier (Axon Instruments, Burlingame, CA, United States). Signals were digitized and analyzed by counting all spikes occurring during the first second of recording using dbWave (Marion-Poll, 1996).

### Metabolite Extraction, Identification and Quantification

For metabolite extraction, 1 g fresh leaf from *P. nigra* (*n* = 6 plants) or *S. viminalis* (*n* = 3 plants) was ground in 3 ml aqueous 80% MeOH using clean mortar and pestle under the addition of liquid nitrogen and acid washed sand (silicon dioxide, 18649 Supelco, Sigma-Aldrich) to maximize homogenisation of plant tissue. After centrifugation at 15,000 *g* for 5 min at 4°C to pellet debris, the supernatant was filtered through a 0,22 μm polyvinylidene fluoride membrane (Durapore^®^, GVWP04700, Merck) by an additional centrifugation step and used for analysis via high pressure liquid chromatography coupled to mass spectrometry (LC-MS) and gas chromatography coupled to mass spectrometry (GC-MS). In the latter case, the filtered supernatant was evaporated in a vacuum centrifuge (Eppendorf^TM^ Concentrator plus F-45-48-1) at 45°C for 30 min and derivatized by adding 50 μl *N*-Methyl-*N*-(trimethylsilyl) trifluoroacetamide and pyridine at 60°C for 30 min. The sample was diluted 1:2 with dichloromethane. LC-MS was carried using an Agilent HP1100 HPLC system equipped with a Purospher^®^ STAR RP-18 endcapped (5 μm) column coupled to a Finnigan^TM^ LTQ^TM^ (Thermo Electron Corporation, Dreieich, Germany) mass spectrometer operated in the atmospheric pressure chemical ionization mode with a vaporizer temperature of 450°C. Samples were analyzed by injection of 5 μl. The gradient elution (90:10) consisted of water with 0.1% formic acid as solvent A and acetonitrile with 0.1% formic acid as solvent B; the final flow rate was 1 ml per minute at a maximal pressure of 230 bar. For identification and quantification of salicin (S0625 Sigma) via a standard curve the formic acid adduct [M + HCOOH - H]^-^ of salicin with m/z 331 was used. GC-MS analysis was used for identification and quantification of sucrose (S0389 Sigma), glucose (G8270 Sigma) and fructose (4981 Roth). It consisted of a ThermoQuest TRACE GC 2000 connected to TRACE MS (Thermo Finnigan) equipped with a ZB5 column and a 10-m guard column (Phenomenex, Torrance, CA, United States). Mass spectra were measured in electron impact mode at 70 eV, 33–450 m/z. Volatiles were eluted under programmed conditions: 40°C (2 min isotherm), followed by heating at 10°C per min to 220°C and at 30°C per min to 280°C, using helium (1.5 ml/min) as the carrier gas. The GC injector (split ratio 1:7), transfer line, and ion source were set at 220, 280, and 200°C, respectively. For each compound, statistic differences of the concentration between poplar and willow leaves were calculated via Mann–Whitney Rank Sum test using SigmaPlot (Systat Software, Erkrath, Germany).

### Feeding Choice Assays

Beetles were starved 24 h before the experiment and allowed to feed for 24 h. Feeding choice assays using leaf disks (cut with a cork borer to a diameter of 13 mm) from *P. nigra* and *S. viminalis* were carried out by placing four disks of each species in an alternating order, mounted on a needle, in a Petri dish (diameter 9 cm) with moist filter paper. One beetle was used per Petri dish (*n* = 5). In addition, baking wafers (wheat flour based, Kuchle Back Oblaten Round, 50 mm) were used as neutral substrate to test feeding initiation triggered by the presence of sucrose or salicin (dissolved in distilled water at 500 mM) in comparison to water controls. Therefore, 50 μl test substance were pipetted onto a wafer divided into eights and put in a plastic box (with fine mesh on lid, 20 cm × 20 cm × 6.5 cm) together with six individual beetles (*n* = 3). Feeding damage on the four leaf disks and wafers per arena was measured digitally using ImageJ (Schneider et al., 2012) by comparing the area before and after the experiment; values were converted into percentage terms. Statistic differences were calculated using *t*-test (including equal variance test) for leaf disk assays or one-way analysis of variance (ANOVA, including equal variance and Shapiro–Wilk normality test) for wafer assays using SigmaPlot 12.

### Weight Gain Assays

*Chrysomela populi* were reared on a no-choice diet using twigs with leaves of either *P. nigra* or *S. viminalis*. Weight was measured regularly for 12 days (excluding the pupal period) from seven beetles per group from the larval to the adult stage in mg. Each individual was kept in a single plastic box (with fine mesh on lid, 10 cm × 10 cm × 6.5 cm); fresh twigs with leaves were supplied daily. Significant differences between the two groups with respect to individual weight gain was analyzed with a two-way repeated measurement analysis of variance (RM-ANOVA, including equal variance and Shapiro–Wilk normality test) using SigmaPlot 12.

## Results

### Sensilla Morphology

**FIGURE 1:**
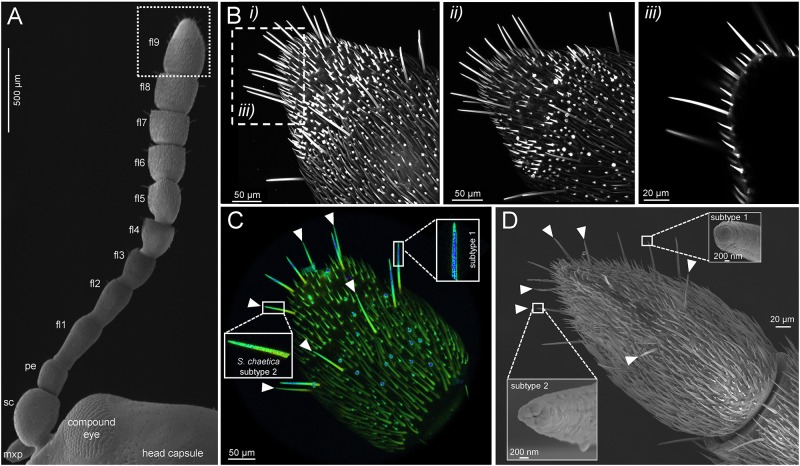
Distinct autofluorescent properties among different antennal sensilla types facilitate identification of gustatory *sensilla chaetica*. **(A)** The antenna of adult *C. populi* consists of scapus (sc), pedicellus (pe) and nine flagellomeres (fl) of which the apical flagellomere was the focus of this study (fl9, dotted frame); mxp - maxillary palp; dorsal view by scanning electron microscopy. **(B)** Cuticular autofluorescence scanning by confocal laser microscopy showing one stack (consisting of 300 scans at 0.5 μm thickness) from the (*i*) dorsal and (*ii*) ventral side; (*iii*) single scan from *i*) as indicated by dashed frame. **(C)** Lambda scan indicates two subtypes of *s. chaetica* due to their distinct fluorescence (subtype 1 in blue; subtype 2 in green with arrowheads). **(D)** Scanning electron microscopy reveals a blunt end of both *s. chaetica* subtypes, but different terminal pore types between subtype 1 and 2 (arrowheads).

The antenna of adult *C. populi* consisted of a scape, a pedicel and a flagellum with 9 subsegments, i.e., flagellomeres (Figure 1A; compare also to Figure 9 in Ge et al., 2015). *Sensilla chaetica* on the 9th and apical flagellomere represented the longest sensilla type with a mean length of 67.8 μm ± 5.8 s.e.m. (Figure 1B). Using autofluorescence scanning, the *s. chaetica* could be distinguished from other sensilla types such as short *sensilla basiconica* or *styloconic* or thin-walled *trichoid* sensilla (Figure 1B). Moreover, there were two subtypes of *s. chaetica* as shown by their distinct fluorescent properties, especially in the more distal parts, resulting in blue (subtype 1) or green (subtype 2) fluorescence as indicated by lambda scanning (Figure 1C). Analysis by scanning electron microscopy showed that the blunt tips of *s. chaetica* subtypes 1 and 2 have different morphologies, especially with respect to their pore types (Figure 1D).

### Confirmation of the Gustatory Function of *Sensilla Chaetica*

**FIGURE 2:**
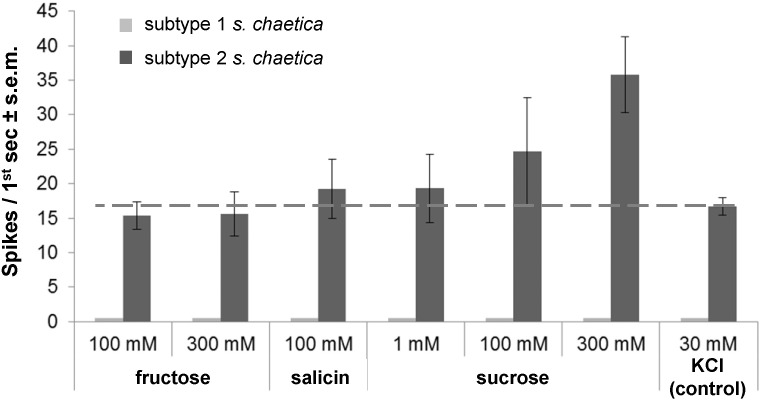
Gustatory receptor neurons in antennal *sensilla chaetica* of subtype 2 respond to salicin, sucrose and salt. Mean spike frequency toward different tastants (dissolved in 30 mM KCl) at different concentrations; 30 mM KCl as control; *n* ≥ 3. Subtype 1 *s. chaetica* did not respond to any of the tastants. Dashed line indicates background activity of gustatory neurons due to 30 mM KCl in tastant solution.

Using single sensillum recordings, the spike activity of GRNs was recorded from *s. chaetica* subtypes 1 and 2 at the antennal tip, i.e., the apical flagellomere (Figure 1A, 2) with sucrose, fructose, salicin and KCl. Using 30 mM KCl that served as positive control and solvent for tastants, mean frequency of *s. chaetica* subtype 2 was 16.7 Hz ± 1.3 s.e.m. (Figure 2). Responses of *s. chaetica* subtype 2 using fructose at 100 and 300 mM were below this control threshold and did not increase with increasing ligand concentration, i.e., 15.3 Hz ± 2.0 s.e.m. and 15.6 Hz ± 3.2 s.e.m., respectively (Figure 2). However, when using sucrose at 1, 100, and 300 mM, spikes from GRNs in *s. chaetica* subtype 2 were on average 19.3 Hz (±5.0 s.e.m.), 24.3 Hz (±7.8 s.e.m.), and 35.8 Hz (±5.5 s.e.m.), respectively (Figure 2). Finally, using salicin at 100 mM, spike discharges were recorded from *s. chaetica* subtype 2 at 19.6 Hz (±2.3 s.e.m.). In contrast, *s. chaetica* subtype 1 did not respond to any of the tested tastants.

### Primary and Secondary Metabolites in Leaves

**FIGURE 3:**
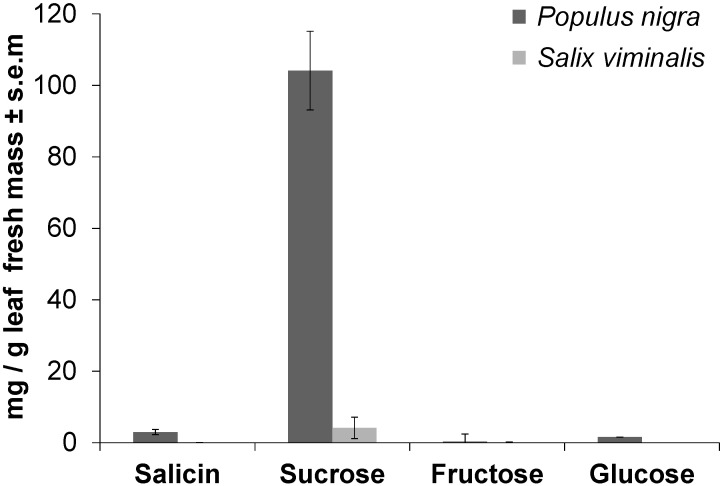
Sucrose and salicin are major compounds in leaves of *Populus nigra*, but less concentrated in *Salix viminalis*. Mean concentration of salicin and different saccharides in leaves of *P. nigra* (*n* = 6) or *S. viminalis* (*n* = 3) as measured by liquid chromatography or gas chromatography coupled to mass spectrometry after extracting fresh leaves in methanol. Authentic standards were used for identification and quantification.

Using LC-MS analysis, methanolic leaf extracts of *P. nigra* were found to contain 3.0 mg (±0.7 s.e.m.) of salicin per g leaf fresh mass, whereas in *S. viminalis* salicin was not detectable (Figure 3). Using GC-MS on the same extracts, sucrose was found to be approximately 25 times higher concentrated in *P. nigra* (104.1 mg ± 11.0 s.e.m. per g leaf fresh mass) than in *S. viminalis* (4.2 mg ± 3.0 s.e.m. per g leaf fresh mass). *P. nigra* leaves contained 1.6 mg (±0.02 s.e.m.) of glucose per gram leaf fresh mass, whereas glucose was not detectable in *S. viminalis* leaves (Figure 3). Each of these three compounds differed significantly between *P. nigra* and *S. viminalis* (*P* < 0.05). Fructose had the lowest concentration in *P. nigra* leaves (0.3 mg ± 0.2 s.e.m. per g leaf fresh mass) compared to the other saccharides tested, whereas in *S. viminalis* fructose was even lower concentrated (0.04 mg ± 0.01 s.e.m. per g leaf fresh mass).

### Feeding Choice and Weight Gain Assays

**FIGURE 4:**
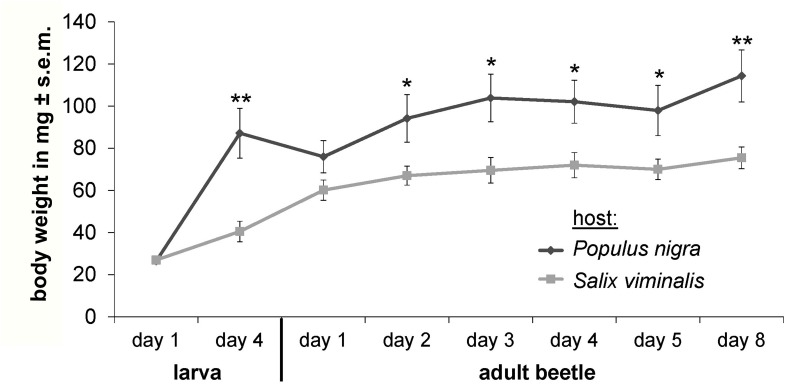
Weight gain of *C. populi* depends on host plant species. Using no-choice diet with leaves from *P. nigra* or *S. viminalis* species shows that individual poplar leaf beetles gain significantly more weight over time on *P. nigra* (*n* = 7) compared to *S. viminalis* (*n* = 7); Two Way Repeated Measurement ANOVA with pairwise multiple comparison (Holm–Sidak method). ^∗^*P* < 0.05, ^∗∗^*P* < 0.01.

Feeding choice assays using leaf disks indicated significant feeding preference of *C. populi* for *P. nigra* over *S. viminalis* (*P* < 0.005); beetles consumed on average 55.3% ± 14.4 s.e.m. of the provided *P. nigra* leaf material, but did not feed on *S. viminalis* (0% feeding damage) if given the choice between these two plant species (Supplementary Figure S1A). Feeding choice assays using baking wafers supplemented with single ligands (sucrose or salicin at 500 mM) indicated significant feeding preference for sucrose (13.0% ± 2.6 s.e.m. feeding damage) over water controls (0.5% ± 0.3 s.e.m.; *P* = 0.03), but not over salicin (7.1% ± 3.2 s.e.m.) (Supplementary Figure S1B). Weight gain assays of *C. populi* using no-choice diets with leaves of *P. nigra* or *S. viminalis* resulted in significantly higher weight gain of those beetles that were reared on *P. nigra* compared to *S. viminalis* leaves (Figure 4).

## Discussion

In phytophagous insects, the main role of the peripheral gustatory system is to provide information about the suitability of plants as food source or oviposition site mainly by sensing compounds that are nutritional, i.e., feeding stimulating, or potentially harmful, i.e., feeding deterrent (Sollai et al., 2014; Pentzold et al., 2017). The plant’s set of primary and secondary metabolites plays a key role in this interaction, since they modulate insect behavior in diverse ways from deterrence to attraction to addiction (Ômura, 2018; Wink, 2018). In turn, insect herbivores sense the majority of non-volatile metabolites via gustation, which requires direct contact with the plant material, finally providing accurate and reliable information about host suitability and identification (Chapman, 2003; Heisswolf et al., 2007; Bustos-Segura and Foley, 2018). In most chrysomelid beetles for example, during antennation of the leaf, gustatory sensilla would be able to detect phytochemicals, present in the surface waxes or the epidermis allowing the beetle to gain information on the suitability of the plant without biting it (Isidoro et al., 1998). However, studying insect gustation at the morphological and physiological level is often hampered by the difficulty of identifying putative gustatory sensilla, because most appendages possess many individual and distinct functional types of sensilla (Hallberg and Hansson, 1999; Faucheux and Kundrata, 2017). To facilitate identification of sensilla involved in contact chemosensation, here we take advantage of the distinct fluorescent properties among the different sensilla types present on the beetle’s antennal tip and confirm their taste responsiveness via electrophysiological recordings using sucrose and salicin. Finally, we show that behavioral choice via contact chemosensation of these cues may contribute to feeding on the host that provides optimal weight gain for poplar leaf beetles.

Autofluorescence scanning of the antennal tips from *C. populi* has identified sensilla of the type *chaetica* (Figure 1C). They are distinct from other sensilla types such as *basiconica*, *styloconic*, and *trichoid* sensilla usually present on a beetle’s antennae (Hallberg, 1982). For example, *s. chaetica* possess the highest amount of cuticular expression, thus they are long, heavy and thick-walled (Ryan, 2002). Similar to other chrysomelid beetle species, *s. chaetica* in *C. populi* were the longest antennal sensilla protruding above all other and are therefore likely involved in contact chemosensation (Isidoro et al., 1998; Zhang et al., 2013, 2016). For example, *s. chaetica* of the cabbage stem flea beetle *Psylliodes chrysocephala* present on terminal flagellomeres respond to host plant-derived taste stimuli such as the non-volatile glucobrassicin (Isidoro et al., 1998). This glucosinolate was also the most effective at stimulating feeding by *P. chrysocephala* (Bartlet et al., 1994). *Sensilla* *chaetica* seem to be the only contact chemosensillar type on the antennae of *P. chrysocephala* (Isidoro et al., 1998) although in the flour beetle *Tribolium brevicornis* a row of small *s. basiconica* on the terminal flagellomere respond to sucrose and NaCl (Alabi et al., 2014). In the lepidopteran *Heliothis virescens* GRNs in antennal *s. chaetica* respond toward sucrose, inositol, salts and bitter substances (Jørgensen et al., 2007). In general, antennal *s. chaetica* in *C. populi* were quite low in their number (14 on the terminal flagellomere) in comparison to other sensilla types, which is similar to other beetle species (Bartlet et al., 1999; Faucheux and Kundrata, 2017).

In some coleopteran species there are two to three distinct subtypes of *s. chaetica* on the antennae depending on the species as shown via electron microscopy (Faucheux and Kundrata, 2017; Zhang et al., 2017). Similarly, there were two subtypes of *s. chaetica* on the terminal antennal flagellomere of *C. populi*, which was revealed in this study using confocal microscopy (Figure 1B,C). Taking advantage of their distinct fluorescent properties by lambda scanning, *s. chaetica* of subtype 1 were identified by their pronounced blue fluorescence, especially in the more distal part, whereas subtype 2 showed green fluorescence equally distributed along the sensilla. The differences in fluorescence between the two subtypes are likely due to different amounts in resilin and chitin, respectively. For example, the adhesive tarsal setae of the ladybird beetle *Coccinella septempunctata* have a pronounced longitudinal material gradient with high concentrations of the elastic protein resilin (blue autofluorescence) in the tips, whereas the less flexible central and proximal parts of the setae mainly consist of other materials such as chitin indicated by green autofluorescence (Peisker et al., 2013). Therefore, subtype 1 *s. chaetica* in *C. populi* could be envisioned as being more flexible than subtype 2 due to higher proportions of resilin, whereas subtype 2 (to agree with the definition of Faucheux and Kundrata, 2017) seems stiffer due to its higher proportion of chitin. These findings further stress the potential of fluorescence and confocal microscopy for visualizing morphological details of external insect structures (Michels and Gorb, 2012) to facilitate the elucidation of distinct material compositions between sensilla subtypes. Furthermore, fluorescence microscopy as described here requires less time for sample preparation than scanning or transmission electron microscopy.

The presence of distinct subtypes of blunt-ended *s. chaetica* in *C. populi* is similar to other beetle species and usually related to a contact chemosensory function (Bartlet et al., 1999; Faucheux and Kundrata, 2017). For example, *s. chaetica* of subtype 3 in elm leaf beetles possess an apical pore and a blunt tip indicating gustatory function (Zhang et al., 2017). Thus, those *s. chaetica* *in C. populi* that were found to possess a clear terminal pore (subtype 2) (Figure 1D), did respond electrophysiologically to host tastants such as sucrose and salicin as well as KCl, whereas subtype 1 did not evoke these responses (Figure 2). The presence of an apical pore is usually characteristic for contact chemosensation, and required for non-volatile plant-derived compounds to enter into the sensillum, dissolve into lymph to reach and stimulate GRNs ultimately mediating behavioral responses (feeding or rejection). Recordings on *s. chaetica* subtype 1 may evoke physiological responses when using other compounds than those tested. Other salicinoids or condensed tannins would be candidates since they are abundantly present in poplar (Barbehenn and Constabel, 2011); this question requires future investigations. That no electrophysiological responses toward fructose could be obtained from *s. chaetica* subtype 2 was consistent with the apparent lack of fructose in the host plant (Figure 3). Thus, fructose is either of little or no importance for sensation in *C. populi* or, alternatively, it is sensed by gustatory *s. chaetica* on mouthparts and tarsi as putatively present in other beetles such as *Tribolium* *castaneum* (Seada and Hamza, 2018). In any case, functional characterisation of single gustatory receptor genes warrants future investigation to reveal the number of putative ligands. In contrast to fructose, sucrose and salicin were highly concentrated in *P. nigra* leaves (Figure 3). Correspondingly, in feeding choice assays *P. nigra* leaves were clearly preferred over *S. viminalis* leaves (Supplementary Figure S1) that contained 25 times less sucrose and lacked salicin. Also other studies indicate that *C. populi* prefer to feed on salicaceous species with relatively high concentrations of phenolic glucosides such as salicin in the leaves (Ikonen, 2002); similarly, adults of *Chrysomela aeneicollis* were stimulated to feed by salicin itself (Rank, 1992). It is conceivable that the metabolic differences between *P. nigra* and *S. viminalis* contributed to the observed significant differences in weight gain over time when *C. populi* beetles were reared on either plant species (Figure 4). Thus, sucrose seems a feeding stimulant, while salicin seems to contribute to feeding initiation, potentially promoting recognition of *P. nigra* leaves by contact and serving increased weight gain during development of *C.* *populi*.

Overall, the combined analysis of autofluorescent properties and morphological details of different sensilla types on the beetle’s antenna facilitated the identification of those sensilla with gustatory function. In case of *s. chaetica* subtype 2 from *C. populi*, beetles physiologically sensed major primary and secondary metabolites from its host plant with their antennal tip via contact chemoreception promoting taste selection of those plant species that provide a nutritional basis for optimal development. However, since the most obvious secondary compounds found in an insect’s host plant may not be the only or even the primary basis of host recognition (Mitchell et al., 1999), future experiments should take into account the structural diversity of (poplar) plant metabolites as well as additional gustatory sensilla likely present on other chemosensory appendages such as mouthparts and tarsi. This will help to elucidate which sensillum responds to which phytochemical and how this influences the insect’s host selection and feeding behavior.

## Data Availability

All datasets generated for this study are included in the manuscript and/or the Supplementary Files.

## Author Contributions

SP and AB contributed conception and design of the study. SP, FM-P, and VG carried out the experiments. SP wrote the first draft of the manuscript. All authors contributed to manuscript revision, read and approved the submitted version.

## Conflict of Interest Statement

The authors declare that the research was conducted in the absence of any commercial or financial relationships that could be construed as a potential conflict of interest.

## Supplementary Material

FIGURE S1 (A) Exemplary two-choice feeding assay using leaf disks of poplar (p) *Populus nigra* and willow (w) *Salix viminalis* indicates highly significant feeding preference of adult *C. populi* for poplar over willow (^∗∗∗^*P* < 0.005; *t*-test); *n* = 5. **(B)** Exemplary feeding choice assay using wafers soaked with sucrose or salicin at 500 mM to test feeding damage (arrowheads) by *C. populi*; *n* = 3. Significant feeding preferences were found for sucrose over water solvent controls (^∗^*P* = 0.03; one-way ANOVA); n.s., not significant.
