# Supplementary figures and images for: Autofluorescence-Based Identification and Functional Validation of Antennal Gustatory Sensilla in a Specialist Leaf Beetle
Source: Front Physiol. 2019 Mar 28;10:343. doi: 10.3389/fphys.2019.00343 (PMC6455084; doi:10.3389/fphys.2019.00343)

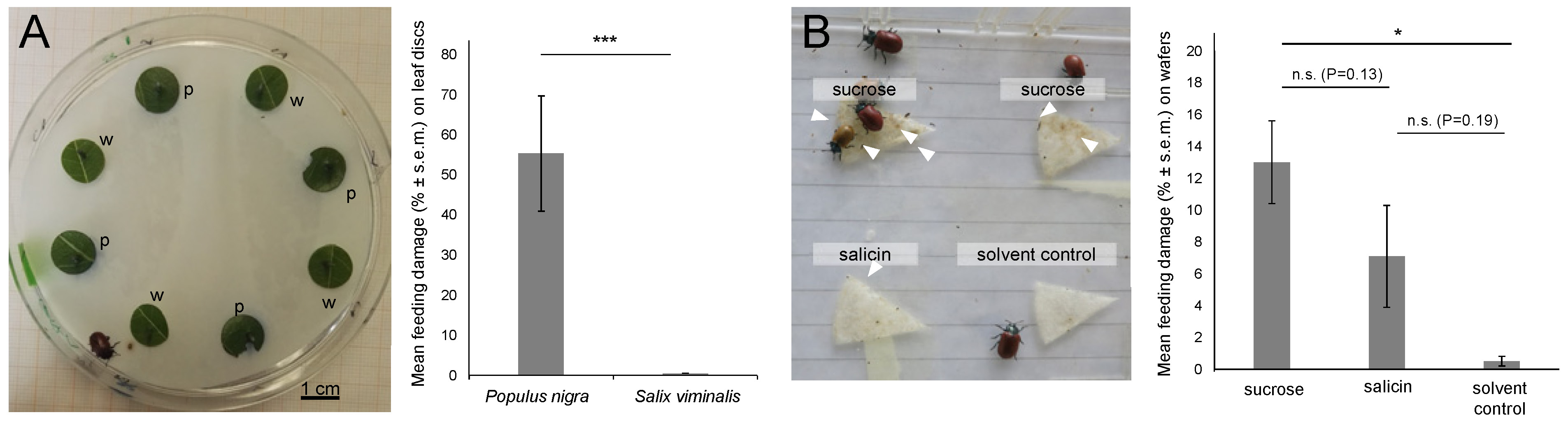

Supplement: FIGURE S1 — (A) Exemplary two-choice feeding assay using leaf disks of poplar (p) Populus nigra and willow (w) Salix viminalis indicates highly significant feeding preference of adult C. populi for poplar over willow (∗∗∗P < 0.005; t-test); n = 5. (B) Exemplary feeding choice assay using wafers soaked with sucrose or salicin at 500 mM to test feeding damage (arrowheads) by C. populi; n = 3. Significant feeding preferences were found for sucrose over water solvent controls (∗P = 0.03; one-way ANOVA); n.s., not significant. [file Image_1.JPEG]
